# Ensuring COVID-19 Vaccines for Migrant and Immigrant Farmworkers

**DOI:** 10.4269/ajtmh.21-0199

**Published:** 2021-04-13

**Authors:** Christine M. Thomas, Amy K. Liebman, Alma Galván, Jonathan D. Kirsch, William M. Stauffer

**Affiliations:** 1 Department of Medicine, Division of Infectious Diseases and International Medicine, University of Minnesota, Minneapolis, Minnesota;; 2 Migrant Clinicians Network, Austin, Texas;; 3 Department of Medicine, Division of General Internal Medicine, University of Minnesota, Minneapolis, Minnesota;; 4 Center for Global Health and Social Responsibility, University of Minnesota, Minneapolis, Minnesota

## Abstract

Migrant and immigrant farmworkers are cornerstones to food security and production in many nations. In the United States, farmworkers have been disproportionately impacted by COVID-19. Because they are considered essential workers, vaccines may be made imminently available to them and offer an opportunity to reduce these COVID-19–related impacts. It is essential for a successful vaccination campaign to address the unique challenges arising from this workforce’s inherently mobile nature and limited access to healthcare. Proposed strategies to overcome these challenges include ensuring farmworkers are prioritized in vaccine allocation and provided cost-free vaccines at convenient locations through partnerships among health authorities, community- and faith-based groups, and health centers with trusted community relationships. Further, a portable immunization record should be used, and coordination of care continued when a farmworker moves to a new geographic location. If implemented well, vaccinating farmworkers can reduce the COVID-19 disease burden among these essential workers, improve public health, and protect food and agriculture production.

Migrant and immigrant farmworkers are integral to the food supply chain globally. In the United States, farmworkers have been disproportionately impacted by COVID-19.^1^ This industry-related impact has been further compounded by COVID-19 health-related disparities and inequities experienced by Latinx and immigrant communities—groups with whom approximately 75% of farmworkers identify.^2^ It is imperative to recognize and mitigate the increased risk of COVID-19 faced by farmworkers because of work-related factors such as shared transportation, high-density housing, close-proximity work environments, barriers to healthcare access, and immigration status. The growing availability of COVID-19 vaccines provides an excellent opportunity to mitigate this risk, if successfully implemented.

Given their integral role and the disproportionate pandemic impact experienced by farmworkers, it is appropriate that they are designated as essential workers eligible for COVID-19 vaccine early in allocation.^3^ However, it is disturbing that many state governments are changing the allocation process. For example, there are changes such as lowering the age eligible for vaccination and opening vaccines to the entire adult public by May 1st prior to providing vaccines to high-priority populations. This change will likely delay vaccine availability for these workers and further vaccine inequity.^4^ Even more concerning is the potential exclusion of many workers based on documentation status. Recently the Governor of Nebraska stated that workers who are not authorized to live and work in the United States would not be eligible for vaccination in his state.^5^ Although his office later amended the comments to refer only to vaccine prioritization, this statement still reflects policy positions that perpetuate health inequality and a profound lack of understanding public health measures needed to end the pandemic.

Farmworkers must be ensured equitable vaccination opportunities and should be prioritized. This population experiences many barriers to successful vaccine implementation, including the following: 1) the workforce’s inherently mobile nature, 2) vaccine-specific factors (e.g., storage requirements, making it physically hard for the vaccine to reach rural locations), 3) workers having a well-founded distrust of government and healthcare organizations (based on historic relationships with immigrant mobile populations), 4) conditions for rampant spread of misinformation through the community, and 5) baseline challenges they have in accessing healthcare because of other existing barriers (e.g., language, cultural factors, health literacy, and cost of medical care related to lack of insurance). Providing vaccination to this population is a monumental task in which logistical obstacles, barriers to healthcare, and prejudices must all be acknowledged and addressed. This commentary describes necessary actions to promote successful vaccination among farmworkers by focusing on assuring legal access to vaccines and administration in a largely mobile workforce that frequently lacks access to traditional methods of healthcare delivery (outlined in Figure 1). Additional considerations for overcoming common barriers stemming from vaccine attitudes, beliefs, and practices have been recently published,^6^ and associated practical resources for implementation are available at the National Resource Center for Refugees, Immigrants, and Migrants website: nrcrim.umn.edu.^7^

**Figure 1.**
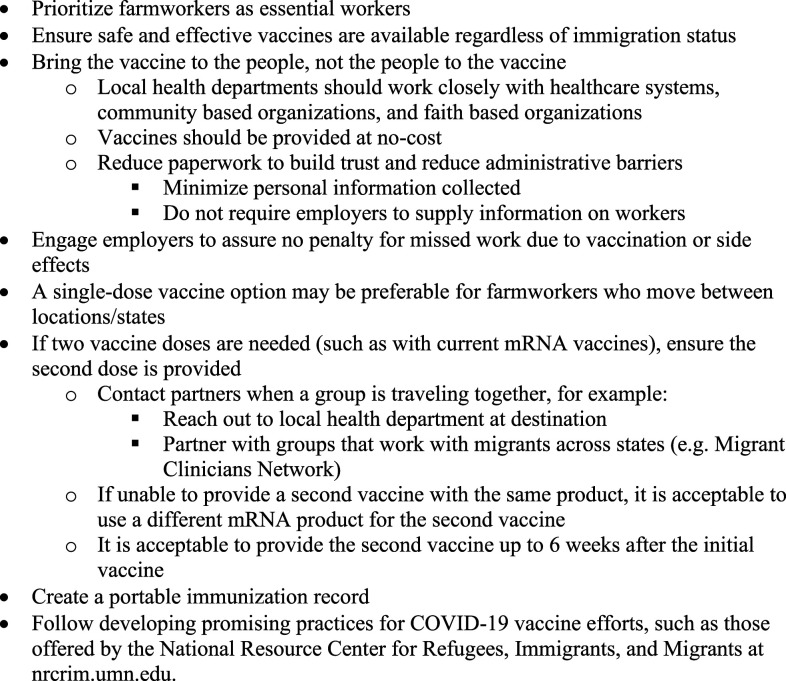
Actions to promote successful vaccination for COVID-19 to farmworkers.

Regarding legal access to vaccines, all people in the United States have been explicitly included in vaccine allocation regardless of legal status or documentation. When published in the fall of 2020, the National Academies Press’s *Framework for Equitable Allocation of COVID-19 Vaccine* specifically stated, “All individuals in the United States and its territories should receive the vaccine in the appropriate phase, irrespective of their legal status.”^8^ More recently, the Biden administration has been clear in the *National Strategy for the COVID-19 Response and Pandemic Preparedness* that there is a federal commitment to “Ensuring safe, effective, cost-free vaccines (are) available to the entire U.S. public—regardless of their immigration status.”^9^ The federal government must back these statements with a commitment to farmworkers; and all states should follow this strategy and ensure vaccination of their entire population, because this task is crucial for public health.

Accessing health care is a challenge for many people in the United States, given the complexity of the healthcare system. This system is extremely challenging for individuals and families who are frequently moving and must overcome existing barriers (e.g., language, transportation, and trust concerns). They must attempt to navigate unfamiliar, changing, confusing, and frequently unwelcoming health systems. These challenges are compounded by cost considerations if they are uninsured, and farmworkers generally receive no worker benefits. In addition, many of these workers are not allowed days away from work. A day away results in pay loss that is significant to families, and time off frequently leads to immediate termination because they are considered replaceable. Faced with this scenario, the expectation that a worker will seek preventative healthcare (vaccination) is unrealistic. To increase the chances of success, vaccines must be brought to the worker rather than relying on the worker to seek the vaccine; and, ideally, the best outcomes will be when employers are educated on the benefits of vaccination for their industry and engaged in vaccine processes.

Although providing vaccination opportunities to farmworkers will be logistically challenging, it is possible with intentional planning. It will also become increasingly feasible with new vaccines, such as the recently available vaccine from Johnson & Johnson, that do not require a second dose or have as stringent storage conditions compared with the initially available mRNA vaccines. Local health departments should provide cost-free vaccines onsite with minimal paperwork requirements to reduce barriers and increase trust. This is best facilitated by working closely with health centers, community-based organizations (CBOs), and faith-based organizations (FBOs) with a history of providing care to hard-to-reach groups. Ideally partnership with employers should occur to help facilitate provision of vaccines at convenient times and locations. Because potential distrust between government, employers, and workers could interfere with vaccine acceptance, trusted community groups should be engaged to help facilitate workplace vaccine clinics. Assurance can be provided that neither worksites nor documentation status will be assessed. Regardless of the employers’ role in providing vaccination, health departments and community partners should engage employers to assure that a worker who cannot return to work because of significant side effects is not punished or terminated. The employer should realize that this strategy is the right public health measure and beneficial economically (e.g., employers don’t need to find and train a new worker, and vaccination will assist in limiting other workers from becoming ill and missing work).

A unique challenge in this population is movement among geographic locations, exacerbated by differing vaccine eligibility and processes in each state. Eligibility differences by location may resolve as vaccine becomes available to the entire U.S. population, but the logistics and recordkeeping of vaccine provision will likely continue to vary by location. For example, although a single-dose vaccine is now available and offers improved convenience, states may differ in which vaccine is available to farmworkers; thus movement between locations poses a barrier to receiving a second dose, if needed. Fortunately, any available mRNA COVID-19 vaccine can be given up to 6 weeks after the initial dose in the setting of exceptional situations,^10^ which offers flexibility—though provision of the vaccine must still be facilitated.

In addition to ensuring vaccines are consistently offered, an accurate immunization record that can be accessed in any geographic location is valuable to avoid miscommunication about vaccine status that could result in missed vaccine opportunities or unnecessary repeated vaccination. Ideally a portable immunization record, like a platform facilitated by Migrant Clinicians Network for COVID-19 vaccines and other health issues,^11^ would be created to assist communication and coordination of care as a worker moves from place to place. In the successful provision of vaccination opportunities, effective communication and care coordination relies on partnerships among health departments, interested employers, and healthcare and community groups (e.g., CBOs and FBOs). Additionally, a federal commitment to ensuring farmworkers have access to the vaccine would greatly benefit from coordination among different states.

This commentary focuses on providing COVID-19 vaccines to farmworkers in the United States, but the challenges of providing vaccines to a mobile workforce are not unique to this country. Migrant workers in essential industries from agriculture, to meat packing, to home healthcare across the globe have been disproportionately impacted by COVID-19.^12^ This problem is further evidenced by an overall COVID-19 infection prevalence greater than 50% among 200,000 migrant workers residing in high-density housing in Singapore.^13^ Providing COVID-19 vaccines to these populations is of utmost ethical importance in the global pandemic response,^14^ and success in ending this pandemic will be heavily weighted on the foresight, planning, and execution of this vaccine provision. Although legal and healthcare systems vary by country, ensuring a legal right and access to COVID-19 vaccines and developing vaccination processes that accommodate worker mobility are universally necessary to reduce the pandemic’s burden on individuals and populations. As such, the strategies presented here can be adapted to suit mobile workers in other settings.

As COVID-19 vaccines are increasingly available, essential workers and those at greatest risk for COVID-19, including farmworkers, must continue to be prioritized. Certainly the mobility of this population poses multiple challenges for vaccination, so resources and planning must be dedicated to ensuring this group receives the opportunity to be vaccinated to promote equitable vaccine access and prevent the further compounding of the COVID-19 burden. Partnering of health authorities with healthcare, industry organizations, CBOs, and FBOs is essential in overcoming these substantial barriers. This method can help protect food and agriculture production, improve the greater public health, and, most importantly, reduce infection, disease, and death in this population that is disproportionately impacted by the pandemic.
